# Importation of a novel Indian Ocean lineage carrying E1-K211E and E2-V264A of Chikungunya Virus in Zhejiang Province, China, in 2019

**DOI:** 10.1007/s11262-023-02020-z

**Published:** 2023-07-19

**Authors:** Lingxuan Su, Xiuyu Lou, Hao Yan, Zhangnv Yang, Haiyan Mao, Wenwu Yao, Yi Sun, Junhang Pan, Yanjun Zhang

**Affiliations:** Zhejiang Provincial Center of Disease Control and Prevention, 3399 Binsheng Road, Hangzhou, 310051 China

**Keywords:** Chikungunya virus (CHIKV), Whole genome sequence, Phylogenetic analysis, Amino acid variation sites, Indian Ocean lineage

## Abstract

The chikungunya virus (CHIKV) is widespread. In Zhejiang province, China, CHIKV infection is often associated with travelers from tropical and subtropical countries. In the present study, three CHIKV isolates from serum samples of travelers in Zhejiang province in 2019 were sequenced, and phylogenetically analyzed to study their molecular characteristics. Sequence analysis showed that the non-structural protein and the structural protein had 37 and 28 amino acid mutations, respectively; no mutation site was found at the E1-A226 residue, which could increase the adaptability of CHIKV to *Aedes albopictus*. All three samples carried two mutations, namely, E1-K211E and E2-V264A, which were introduced to Bangladesh around late 2015 and Thailand in early 2017. Phylogenetic analysis revealed that these three CHIKVs were Indian Ocean lineage of the East Africa/Central/South Africa genotype (ECSA) and that the MF773566 strain from Bangladesh (Australia/Bangladesh 2017) had the closest evolutionary relationship. The three CHICKs imported into Zhejiang province in 2019 belonged to the ECSA genotype and had multiple amino acid variation sites. The variation in the three samples provides a certain reference for the subsequent research on CHIKV evolution.

## Introduction

Chikungunya fever is a disease caused by the chikungunya virus (CHIKV) primarily transmitted by *Aedes aegypti* and *Aedes albopictus*, which has undergone widespread geographic expansion in the past 15–20 years. Approximately 3–5 million cases of CHIKV are reported globally every year [1]. CHIKV was first characterized in 1952 during an outbreak in the Newala District of Tanzania [1]. Starting in Africa, more than 110 countries and territories over tropical and subtropical areas spanning Africa, Asia, the Americas, and Europe have reported the autochthonous transmission of CHIKV [2–7].

CHIKV belongs to the genus *Alphavirus* in the family *Togaviridae* and is a small enveloped virus with a linear, positive-sense single-stranded RNA genome of approximately 11.8 kb. The genome of CHIKV consists of two open reading frames (ORFs) flanked by 5’ and 3’ non-translated regions. The positive sense 5’ RNA genome directly encodes one polyprotein containing four non-structural proteins (nsP1, nsP2, nsP3, and nsP4), and the structural polyprotein is encoded by 3’ RNA genome and converted into a capsid glycoprotein (C), two major envelope surface glycoproteins (E1 and E2), and two small peptides (E3 and 6 K). Phylogenetically, CHIKV consists of three major genotypes, namely, the West African genotype, the East Central South African (ECSA) genotype including the Indian Ocean lineage (IOL), and the Asian genotype based on viral genome sequences and evolutionary analysis [8].

Although mortality due to CHIKV infection is rare, it can cause three acute clinical forms (acute, atypical acute, and severe acute) and a chronic form depending on the manifestation of symptoms in humans, as defined by the World Health Organization (WHO) [9]. The main clinical symptoms of chikungunya are the rapid onset of high-grade fever, frequently accompanied by arthralgia, primarily of the peripheral joints [10]. Other common signs and symptoms include myalgias, joint swelling, headaches, nausea, severe fatigue, and maculopapular skin [11]. However, several clinical symptoms of CHIKV infection overlap with those of dengue virus (DENV) and Zika virus (ZIKV) infections [12].

CHIKV was first detected in South Asia a shortly after its identification in East Africa. Since the 1960s, several disastrous epidemics or small epidemics have occurred in South Asia and Southeast Asia [6], especially after 2007; CHIKV outbreaks were larger and longer and infected millions of people in India [13–16], Thailand [17], and Bangladesh [18]. In China, CHIKV infection was first reported in Yunnan province in 1986, and local outbreaks caused by the imported cases have been documented in Guangdong [19] and Zhejiang provinces [20]. Furthermore, imported CHIKV cases have also been found in most regions of South China, such as Anhui province, Shanghai, Sichuan province, and Guizhou province recently [21]. Therefore, to better control the epidemics of CHIKV, it is necessary to understand the evolutionary and variational characteristics of CHIKV. Accordingly, in our study, 3 chikungunya fever cases were used to conduct a molecular and phylogenetic characterization of CHIKV that were detected in Zhejiang province in 2019.

## Materials and methods

### Sample collection

Three imported cases of chikungunya fever were reported in Zhejiang province in 2019. CHIKV nucleotide detection was performed according to the diagnostic criteria for CHIKV (WS/T 590–2018) of the National Health Commission of the People’s Republic of China. The first and second cases (ZJJX19-1 and ZJJX19-2) were found in patients travelling to Thailand, their onset dates were July 10 and July 11, respectively, both sampled on July 12. The third case (ZJHZ19-3) was found in a patient returning from Myanmar; the onset date was July 31 and the sample was collected on August 1. In 2019, chikungunya fever outbreaks were reported in both Thailand and Myanmar, especially in Thailand, where more than 10,000 cases were exhausted in 2018–2019 [22, 23].

### Reverse transcription-polymerase chain reaction (RT-PCR)

The RNA from patient serum samples was extracted using the RNeasy Mini Kit (Qiagen, Germany). Real time RT-PCR was conducted using the One Step PrimeScript™ RT-PCR Kit (TaKaRa, Japan) and the following primers and probe: CHIKV-FP (5’-TTTAGCCGTAATGAGCRTCGG-3’), CHIKV-RP (5’-CCGTGTTCGGGATCACTGTTA-3’) and CHIKV-P (5’-FAM-TGCCCACACTGTGA–BHQ1-3’). The 25 µL PCR reaction mixture included 12.5 µL of 2 × One Step RT-PCR buffer III, 0.5 µL of Ex Taq HS (5 U/µL), 0.5 µL of PrimeScript RT Enzyme Mix II, 0.6 µL of each primer (20 µM), 0.3 µL of probe (20 µM), 5 µL template RNA and 5 µL RNase Free dH_2_O. The following thermal profile was performed on Applied Biosystems 7500 using a single cycle of reverse transcription for 15 min at 42℃, 2 min at 95℃ for reverse transcriptase inactivation, and DNA polymerase activation followed by 45 amplification cycles of 10 s at 95℃ and 30 s 60℃ (annealing-extension step). Data were analyzed by Applied Biosystems software.

### Whole genome sequencing and phylogenetic analysis

For each viral RNA sample, a random primer cDNA library was initially prepared, and the quality of cDNA preparation was monitored by using the standard Illumina protocol. The libraries were sequenced using the MiSeq reagent kit v3 and Illumina MiSeq system. Raw data, contig assembly, alignment to the reference sequence, and comparative analyses were carried out using CLC Genomics Workbench 12.0 (QIAGEN, CA, USA); as the first discovered strain of CHIKV, the complete genome sequence of the S27 African prototype (AF369024) was used as the reference sequence.

GeneDoc v2.7 and the Lasergene suite of programs (DNASTAR, Madison, USA) were used to analyze the nucleotide and deduced amino acid sequence identities [24].

From the GenBank database, a total of 88 reference sequences of CHIKV from 1953 to 2020 were retrieved and downloaded. In this study, three nucleotide sequences of CHIKV and 88 sequences were aligned using the ClustalW method in MEGA v11.0.10 [25]. The bestfit nucleotide substitution model was determined using MEGA models. MEGA v11.0.10 was used in the phylogenetic analysis to sequence the complete coding region using the maximum-likelihood method (ML) in the Kimura 2 parameter model of gamma distribution rates with 1000 bootstrap replicates.

### Bayesian evolutionary analysis

Using the BEAST package v.1.8.4 with Markov Chain Monte Carlo algorithms (MCMC) [26], a total of 91 CHIKV sequences were used to estimate the nucleotide substitution rates and the time to the nearest common ancestor (TMRCA), meanwhile, the input file for BEAST was created by BEAUti v1.8.4. The general time reversible (GTR) method with gamma distribution (G) was selected as the best-fit site rate variation model (GTR + G4) for phylogenetic analysis with the partial E1 gene dataset.

The best model of a relaxed uncorrelated lognormal molecular clock with a Bayesian skyline coalescent was used to evaluate the dataset. For the BEAST analysis, it mainly runs twice for 100,000,000 generations, and sample the parameter values after each 10,000 steps. The combined result of the log files with an effective sample size (ESS) greater than 200 was analyzed and viewed using Tracer v1.7.1 (http://tree.bio.ed.ac.uk/software/tracer/); the maximum clade credibility (MCC) tree was constructed using TreeAnnotator v1.8.4 and visualized using the FigTree v1.4.4 program.

### Selection pressure analysis

The selection pressure was estimated for 91 partial E1 gene sequences via four methods using the Datamonkey online server (www.datamonkey.org)[27]. Sequences with ambiguous results and high similarity (99% identity) were automatically excluded from the selection pressure analysis. Estimation of dN/dS (ratio of non-synonymous to synonymous mutations) was performed using four different approaches-mixed effects model of evolution (MEME), single-likelihood ancestor counting (SLAC), fast unconstrained bayesian approximation (FUBAR), fixed effects likelihood (FEL), methods.

### Shannon entropy analysis

Shannon entropy analysis was performed using BioEdit v7.2.5 software to identify highly variable sites in the amino acid sequences with high entropy values. The cut-off value of 0.2 was set, and sites with values > 0.2 were considered variables [28].

### Ethics statement

All methods in this study were approved the Institutional Ethics Committee (Zhejiang Provincial Center for Disease Control and Prevention).

## Results

### CHIKV RT-PCR and sequencing

The nucleotides of ZJJX19-1, ZJJX19-2, and ZJHZ19-3 were amplified from serum RNA extract by RT-PCR, and confirmed to be CHIKV-positive. The genome sequences of the three CHIKV samples were successfully sequenced and deposited in the GenBank database with the following accession numbers: OL840905, OL840906, and OL840907 for ZJJX19-1, ZJJX19-2 and ZJHZ19-3, respectively.

### Sequences analysis

Consistent with the CHIKV reference sequence, the genomes of three samples contained two independent ORFs, one encoding structural proteins (C, E3, E2, 6K and E1), and the other encoding non-structural proteins (nsP1, nsP2, nsP3, and nsP4). The sequence lengths of ZJJX19-1, ZJJX19-2, and ZJHZ19-3 were 11,786 bp, 11,786 bp, and 11,436 bp, respectively, and the GC contents of these sequences were 50.2%, 50.2%, and 50.68%, respectively.

There was only one nucleic acid base difference between ZJJX19-1 and ZJJX19-2 genome sequences in the nsP1 region, and six different nucleic acid bases were found between ZJJX19-1 and ZJHZ19-3 in the consensus sequence. Further similarity analysis via BLAST showed that the strains most similar to these samples were 19RL50 (2019) (MW110476, 99.97%), isolated from Yunnan, China in 2019. Three strains from Thailand (MN974219, MN630017, and MN974204), they showed shared 99.90-99.96% nucleotide identity with ZJJX19-1.

Compared with those in the reference strain S27 African prototype (GenBank accession No. AF369024), there were 30, 31, and 31 amino acid substitutions in the ZJJX19-1, ZJJX19-2, and ZJHZ19-3 non-structural proteins of CHIKV, respectively; 21 identical amino acid substitutions in the structural proteins were found in these three samples.

Compared to the strain S27 African prototype, the substitutions occurred mainly in the nsP1, nsP2, nsp3, nsP4, and E2 proteins of these three samples. However, the 6K protein was relatively conserved and no amino acid substitutions were found. In addition, fewer amino acids substitutions were found in the C, E3, and E1 proteins. The most variable regions in the three CHIKV samples were identified in the E2 protein.

Compared with those in the strain S27 African prototype, many of the amino acid substitutions in the ZJJX19-1 were conserved and mainly consisted of the exchange of amino acids with similar physicochemical properties; however, few amino acids mutations were involved in changes in polarity, such as E2-A164T: A (Ala) →T (Thr), nsP3-I376T: I (Ile) →T (Thr), and nsP4-T254A: T (Thr) →A (Ala). Moreover, these isolates shared unique amino acid substitutions at certain sites. The substitution profiles were presented in Table 1.

**Table 1:** Amino acid differences between the S27 and CHIKV samples

Protein	Regions	Amino acid differences between S27 and ZJJX19-1	Amino acid differences between S27 and ZJJX19-2	Amino acid differences between S27 and ZJHZ19-3
Non-structural region	nsP1	T128K, E234K, T376M, T481I, Q488R, L507R	A17T, T128K, E234K, T376M, T481I, Q488R, L507R	T128K, E234K, T376M, T481I, Q488R, L507R
	nsP2	S54N, H130Y, E145D, H374Y, N495S, C642Y, S643N	S54N, H130Y, E145D, H374Y, N495S, C642Y, S643N	S54N, H130Y, E145D, H374Y, N495S, C642Y, S643N
	nsP3	V175I, Y217H, P326S, V331A, T337I, K352E, D372E, I376T, A382T	V175I, Y217H, P326S, V331A, T337I, K352E, D372E, I376T, A382T	V175I, Y217H, P326S, V331A, T337I, K352E, D372E, I376T, A382T
	nsP4	S55N, T75A, R85G, T254A, Q500L, I514T, V555I, V604I	S55N, T75A, R85G, T254A, Q500L, I514T, V555I, V604I	S55N, T75A, R85G, T254A, M487V, Q500L, I514T, V555I, V604I
Structural region	C	P23S, V27I, K63R, K73R	P23S, V27I, K63R, K73R	P23S, V27I, K63R, K73R
	E3	I23T	I23T	I23T
	E2	G57K, G79E, N160T, A164T, S194G, G205S, I211T, V264A, M267R, S299N, T312M, A344T, V386A	G57K, G79E, N160T, A164T, S194G, G205S, I211T, V264A, M267R, S299N, T312M, A344T, V386A	G57K, G79E, N160T, A164T, S194G, G205S, I211T, V264A, M267R, S299N, T312M, A344T, V386A
	E1	K211E, D284E, V322A	K211E, D284E, V322A	K211E, D284E, V322A

Amino acid differences were reported following the alignment of sequences obtained from the samples in this study (ZJJX19-1, ZJJX19-2, and ZJHZ19-3). The first amino acid named was found in S27, and the second amino acid was found in the samples

### Phylogenetic analysis

**Fig. 1:**
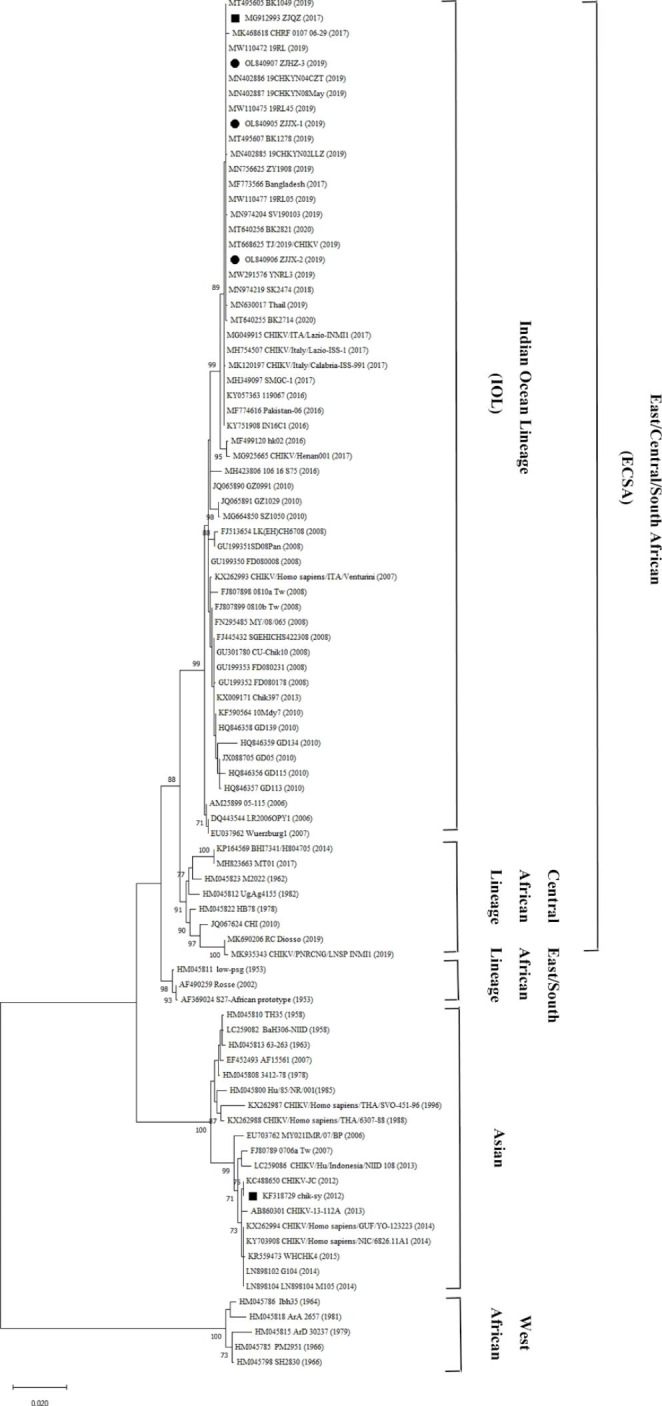
Phylogenetic tree generated by maximum likelihood analysis of the full-coding regions of CHIKV constructed using MEGA v11.0.10 software. Three CHIKV samples in this study are indicated with ●. “Accession number”, “Name” and “Year” were used to indicate the representative strains of each genotype obtained from GenBank. MG912993 and KF318729 indicated with ■ were the strains that caused outbreaks in Zhejiang Province in 2017 and 2012, respectively

To determine the evolutionary history of these three samples, phylogenetic tree was constructed based on the E1 genes of CHIKV. Phylogenetic analysis of 91 CHIKV strains based on the full coding regions (Fig. 1) revealed that all of these three samples (ZJJX19-1, ZJJX19-2, and ZJHZ19-3) belonged to the IOL in the ECSA genotype. They were closely related to contemporaneous CHIKV strains from Southeast Asian, such as the isolates in Thailand (MN630017, MN974204, MT495607, and MT640255), and strains isolated from patients in Tianjin (MT668625) and Zunyi (MN756625), Guizhou province, China who returned from Myanmar in 2019. Interestingly, several isolates (MW110472, MN402887, and MW291576) identified in Yunnan province, China in 2019 also belonged to IOL.

### Bayesian evolutionary analysis

**Fig. 2:**
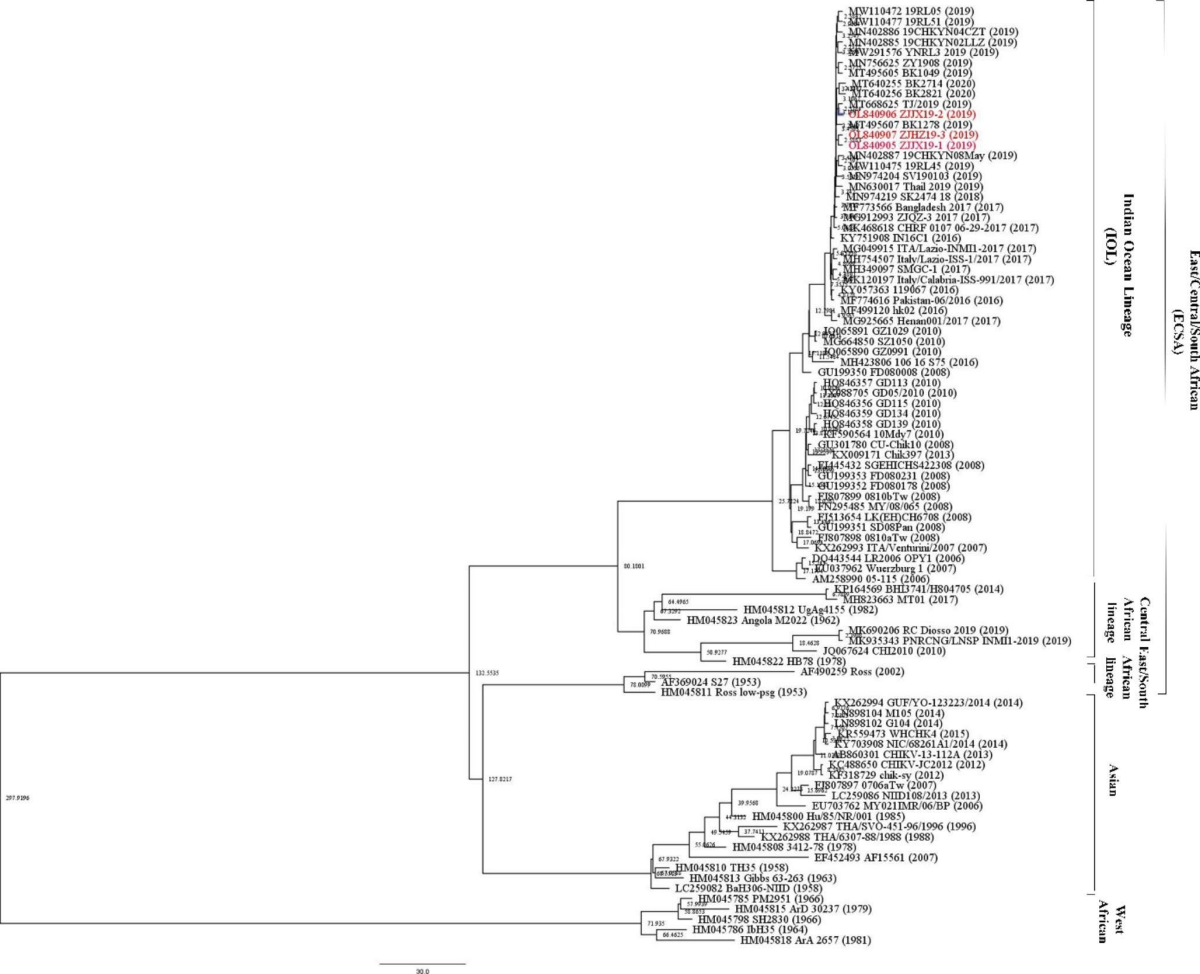
Bayesian evolutionary analysis of CHIKV in this study. Red-marked strains indicate the CHIKV strains isolated in this study. The nodes indicate the node heights

The Bayesian skyline coalescent tree prior to the relaxed clock was selected to be the best fit based on the Bayes factor. The maximum clade credibility tree was constructed using the log file generated in this run (Fig. 2); the MCMC tree also revealed the clustering pattern of the sequences, as described earlier using phylogenetic analysis. For the 91 E1 genes of CHIKV strains, the rates of nucleotide substitution and tMRCA with over 200 ESS values were obtained. The CHIKV was calculated to have originated around 298 years ago (1721), with 95% highest posterior density (HPD) ranging from 108 (1911) to 558 years (1461) with a nucleotide substitution rate of 3.95 × 10^− 4^ (HPD 95%: 2.19 × 10^− 4^ to 5.98 × 10^− 4^) across all the genotypes in this study.

### Selection pressure and recombination analysis

Table 2 summarized the results of the selection pressure, which were analyzed with normalized dN/dS. The low dN/dS ratio (range is 0.065–0.070) suggested purifying selection in this region of the E1. This dataset consisted of 59 E1 gene sequences of CHIKV (32 sequences were excluded for high similarity). No positive selection was observed using the SLAC method in this dataset, but 46 negatively selected sites were observed with a value of 0.1 (data not shown). Four amino acid sites were found to be positively selected by FEL (145, 211, 321, and 377), and three positively selected sites (145, 211, and 377) were identified using the FUBAR method. Nineteen positively selected sites were observed using MEME (4, 19, 28, 145, 157, 195, 196, 211, 235, 250, 275, 316, 321, 341, 351, 377, 382, 391 and 397). Three amino acid positions (145, 211, and 377) were found to be under positive selection using three methods, and one amino acid (321) using two different methods. Significantly, mutations in position 211 were found in most strains of all three genotypes. Some of these mutations were genotype-specific.

**Table 2:** **A**nalyses of selection pressure of CHIKV *E1* gene

Nucleotide substitution methods						
	SLAC		MEME		FEL	FUBAR
*p* value	dN/dS	Positive sites	dN/dS	Positive sites	Positive sites	Positive sites
0.10	0.070	N	0.065	145, 316, 341, 377, 382	145	211, 377
0.15	0.070	N	0.065	145, 157, 211, 235, 316, 341, 377, 382	145, 317	145, 211, 377
0.20	0.070	N	0.065	28, 145, 157, 211, 235, 250, 275, 316, 341, 377, 382	145, 211, 377	145, 211, 377
0.25	0.070	N	0.065	4, 19, 28, 145, 157, 195, 196, 211, 235, 250, 275, 316, 321, 341, 351, 377, 382, 391, 397	145, 211, 321, 377	145, 211, 377

SLAC: Single-Likelihood Ancestor Counting; MEME: Mixed Effects Model of Evolution; FEL: Fixed Effects Likelihood; FUBAR: Fast, Unconstrained Bayesian AppRoximation.

To search for any potential recombination events in CHIKV that could affect the phylogenetic structure, an exhaustive, triplet-by-triplet screening of genomic sequences was performed in RDP version 4.101 with a set of different recombination detection methods, including RDP, Chimaera, GENECONV, BootScan, MaxChi, SiScan, and 3 Seq. No evidence of recombination was observed in this dataset.

**Fig. 3:**
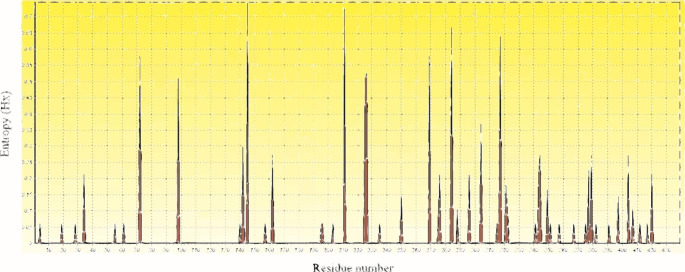
Shannon entropy analysis plot showing entropy values of different amino acid residues. The higher entropy value depicts an enhanced probability of being a variable site

### Shannon entropy analysis

Shannon entropy analysis of the protein sequences of E1 showed variable sites in this study (Fig. 3). An entropy value above the threshold of 0.2 indicates the presence of variations at these sites. A total of 21 variable sites with entropy values higher than 0.2 were selected at the amino acid positions 34, 72, 98, 142, 145, 162, 211, 225, 226, 269, 276, 284, 296, 304, 327, 343, 344, 377, 379, 404, and 420 with entropy values higher than 0.2. The positions numbers 145, 211, 284, and 317 have high entropy values (≥ 0.6).

## Discussion

Due to global warming, the increase in global travel and the expansion of urbanization, there has been a continuous increase in chikungunya fever outbreaks worldwide, and the risk of local transmission of CHIKV in areas where there has been no virus epidemic is increasing [5, 29–33]. CHIKV infections have increasingly been detected in Chinese travelers returning from CHIKV-endemic countries, and have even caused the local outbreaks [34, 35], such as those experienced in Guangdong (2010) and Zhejiang (2017), respectively, making it an important public health concern in China. However, information about CHIKV isolates from China is limited, and disease surveillance and prevention employing genome sequencing and molecular evolutionary studies of CHIKV isolates are of great importance. In this report, we present a molecular and evolutionary analysis of CHIKV strains isolated from three overseas travelers in Zhejiang Province, China, in 2019.

In the present study, three strains (ZJJX19-1, ZJJX19-2, and ZJHZ19-3) were detected in the sera of three patients, and whole genome sequencing was performed to analyze the molecular characteristics and genetic relationship of these strains. In phylogenetic analysis, three samples fell into IOL in the ESCA genotype based on genome sequences (Fig. 1), and clustered with the sequences from Thailand, Bangladesh, Myanmar, and China, and the high genome similarity with these sequences confirmed the close genetic relationship with contemporaneous epidemic CHIKV strains from Southeast Asian. Since 2004, the IOL lineage has caused the most CHIKV outbreaks in South Asian, and is transmitted rapidly in this region [4, 14, 29]. From 2010 to 2020, ECSA-IOL strains were associated with the outbreaks in Bangladesh, India, Bhutan and Si Lanka [6, 18, 29], and CHIKV outbreaks in the Guangdong and Zhejiang provinces of China in 2010 and 2017, respectively, were also caused by imported ECSA-IOL strains [19, 20]. These results indicated that the predominant type of ECSA-IOL is circulating in South Asia and Southeast Asia [4–6, 14].

In this study, three isolates shared almost the same amino acid substitution profile, and some amino acid variations in both non-structural proteins and structural proteins were found when compared to the reference strain S27African prototype, however, two variations, nsP1-A17T and nsP4-M487V were found only in ZJJX19-2 and ZJHZ19-3, respectively. In the non-structural protein of the viral helicase domain, two significant amino acid variations, S54N and H374Y, in nsP2 were observed in this region. Mutations in the present strains, including H130Y and E145D in nsP2, R85G in nsP4, K73R in C, and G205S in E2 were also found in other ECSA genotype CHIKV isolates from post-2015 outbreaks in South and Southeast Asia, including Bangladesh, Pakistan, India, and Thailand [4, 14, 17, 18, 29].

The mutation E1-A226V found in IOL-ECSA sequences has contributed to the evolutionary success of this emerging IOL-ECSA strains in the novel urban vector *Ae. albopictus*, which is responsible for increased midgut infectivity, dissemination, and transmission by *Ae. albopictus* but had little effect on *Ae. aegypti* infection; however, E1-A226V was not present in our isolates. It has been demonstrated that of the adaptive mutations in previously reported E2 envelope protein [36], such as E2-60D and E2-I211T, which were present in our samples, E2-G60D increases the infectivity in *Ae. albopictus* and *Ae. aegypti*, regardless of the amino acid (alanine or valine) at position E1-226. By contrast, E2-I211T increased the infectivity in *Ae. albopictus* of strains CHIKV E1-226 V but did not affect *Ae. aegypti*.

Recently, the most outbreaks of IOL-ESCA during 2010 in India [37] and 2016–2017 in India, Pakistan, and Bangladesh also lacked E1-A226V [4, 18]. Instead, they carried two novel mutations: E1-K211E and E2-V264A. Notably, these mutations were also present in these strains. According to a study by Ankita Agarwal et al., CHIKV containing E1-K211E and E2-V264A mutations with E1-226 A displayed remarkably higher fitness for *Ae. aegypti* with a significant increase in virus infectivity (13 fold), dissemination (15 fold) and transmission (62 fold) compared to the parental E1-226 A virus [38]. RNA viruses have a high degree of genetic diversity, and their high mutation rate and quasi species dynamics make them have highly adaptive potential. These mutations may be responsible for the rapid spread of CHIKV by increasing its prevalence in *Ae. albopictus* or *Ae. aegypti*. By facilitating the continued expansion of the virus to urban centers and areas with more temperate climates, however, *Ae. albopictus* and *Ae. aegypti* widely inhabit in Southeast Asia and Southern China [6, 39].

Evidence of CHIKV was suggested to occur in different regions as early as the 1770s [40], and the dataset in this study also supported that the CHIKV infection in humans originated around 298 years ago (1721). This tMRCA is similar to the reports that described the origin of CHIKV as 300–500 years ago [13]. Evidence of selection pressure was found in the E1 gene of CHIKV by different methods of calculation, three of amino acid positions 145, 211, and 377 were positively selected and 211 had high Shannon entropy, suggesting higher chances of mutation at these sites. However, a potential recombination that can affect phylogenetic structure, and genomic sequences was not found in this dataset. The biological function of substitutions in these CHIKVs is unknown, and it would be of great significance to carry out future studies to understand how these changes in the genome influence the pathogenesis and transmission of CHIKV.

Although CHIKV is not known to have established an endemic transmission in Zhejiang, a suitable vector for *Ae. Albopictus*, and subtropical climates suggest the potential for repeated introductions and epidemics. However, the spectacular global expansion of CHIKV since 2004, and the spread of the mosquito vectors *Ae. aegypti* and *Ae. albopictus*, indicates that CHIKV will continue to expand its global distribution and become an increasing threat to public health [39]. In addition, the majority of CHIKV cases have been reported in the Southeast Asian countries, such as Thailand, Malaysia, and Myanmar, which are the popular destinations for Zhejiang travelers. These Southeast Asian countries are also endemic areas for dengue fever, which presents with clinical symptoms very similar to chikungunya. The continuous evolutionary variation of CHIKV has the potential to cause the original detection method to fail to specifically detect CHIKV; therefore, it is desirable to have a more comprehensive molecular characterization of CHIKV. Because of the COVID-19 pandemic, international travelers are fewer than before, but once the COVID-19 pandemic subsides, or immigration policies change, the challenges posed by CHIKV will be unprecedented, and we should prepare to deal with CHIKV. Our study can contribute to further research on the transmission of CHIKV, by drawing attention to CHIKV, and strengthening disease surveillance.

## Data Availability

The data presented in this study are available in this paper.
